# Mode of prostate cancer detection is associated with the psychological wellbeing of survivors: results from the PiCTure study

**DOI:** 10.1007/s00520-015-3033-x

**Published:** 2015-11-23

**Authors:** Frances J. Drummond, Eamonn O’Leary, Anna Gavin, Heather Kinnear, Linda Sharp

**Affiliations:** 1 National Cancer Registry Ireland, Building 6800, Airport Business Park, Kinsale Road, Cork, Ireland; 2 Northern Ireland Cancer Registry, Centre for Public Health, Queen’s University Belfast, Mulhouse Building, Grosvenor Road, Belfast, BT12 6BJ Ireland; 3 University of Newcastle, Tyne and Wear, Newcastle upon Tyne, NE1 7RU UK; 4 School of Nursing and Midwifery, University College Cork, Cork, Ireland

**Keywords:** Prostate cancer, Depression, Anxiety, Stress, Prostate specific antigen, PSA, Screening

## Abstract

**Purpose:**

Many men with prostate cancer are asymptomatic, diagnosed following prostate specific antigen (PSA) testing. We investigate whether mode of detection, i.e. ‘PSA detected’ or ‘clinically detected’, was associated with psychological wellbeing among prostate cancer survivors.

**Methods:**

A cross-sectional postal questionnaire was administered in 2012 to 6559 prostate cancer (ICD10 C61) survivors up to 18 years post-diagnosis, identified through population-based cancer registries in Ireland. Psychological wellbeing was assessed using the Depression Anxiety Stress Scale-21. Logistic regression was used to investigate associations between mode of detection and depression, anxiety and stress, adjusting for socio-demographic and clinical confounders.

**Results:**

The response rate was 54 % (3348/6262). Fifty-nine percent of survivors were diagnosed with asymptomatic PSA-tested disease. Prevalence of depression (13.8 vs 20.7 %; *p* < 0.001), anxiety (13.6 vs 20.9 %; *p* < 0.001) and stress (8.7 vs 13.8 %; *p* < 0.001) were significantly lower among survivors diagnosed with PSA-detected, than clinically detected disease. After adjusting for clinical and socio-demographic factors, survivors with clinically detected disease had significantly higher risk of depression (odds ratio (OR) = 1.46 95 % CI 1.18, 1.80; *p* = 0.001), anxiety (OR = 1.36 95 % CI 1.09, 1.68; *p* = 0.006) and stress (OR = 1.43 95 % CI 1.11, 1.85; *p* = 0.006) than survivors with PSA-detected disease.

**Conclusions:**

These findings contribute to the ongoing debate on benefits and risks of PSA testing and may be considered by policy makers formulating population-based prostate cancer screening policies. The relatively high prevalence of negative psychological states among survivors means that a ‘risk-adapted approach’ should be implemented to screen survivors most at risk of psychological morbidity for psychological health, and mode of detection could be considered as a risk stratum.

**Electronic supplementary material:**

The online version of this article (doi:10.1007/s00520-015-3033-x) contains supplementary material, which is available to authorized users.

## Introduction

Screening is a balance between benefit and risk [1]. Widespread use of prostate specific antigen (PSA) testing for prostate cancer detection in general practice has stimulated interest in whether population-based prostate cancer screening programmes should be implemented [2–4]. Although PSA testing has resulted in a decrease in cancer stage and grade at detection [5, 6], its benefits regarding prostate cancer mortality remain unresolved, with contradictory findings from two randomised controlled trials, after 13 years follow-up [7, 8]. Furthermore, PSA testing carries substantial risks including over-diagnosis and over-treatment [7, 9]. Screening may also be associated with the risk of adverse psychological effects; PSA testing has been shown to adversely affect men’s short-term psychological health by increasing prostate cancer-related worry [10, 11]. Additionally, prevalence of depression and anxiety is higher among men with prostate cancer than age-matched controls without prostate cancer [12, 13]. However, the effect of being diagnosed with asymptomatic prostate cancer via PSA testing on the psychological wellbeing of survivors has not, to our knowledge, been investigated.

To inform rational decision-making around PSA testing, it is essential to have a comprehensive understanding of the benefits and risks. To contribute to this understanding, we aimed to investigate whether mode of detection (asymptomatic PSA detected vs symptomatic clinically detected) was associated with the psychological health of prostate cancer survivors, in a large, population-based study.

## Methods

### Setting

The Republic of Ireland (RoI) has a mixed public-private healthcare system, and approximately half the population has private health insurance. Northern Ireland (NI) has a primarily public health system. Prostate cancer screening is not recommended in either jurisdiction. The RoI had no national guidelines until 2011, when the National Cancer Control Programme recommended that men with a raised PSA have a repeat PSA test after 6 weeks, followed by a biopsy referral if the level remains raised [14]. The National Institute for Health and Care Excellence (NICE) prostate cancer detection guidelines (2008) advocate a repeat PSA test after 7 weeks among men in NI [15]. There was evidence of unregulated, opportunistic case finding in the RoI (1994–2005), PSA screening in NI (1990–1999) and higher rates of PSA testing in the RoI compared to NI [4, 6].

### Participants

Methods from the PiCTure study were described previously [16]. Briefly, a population-based sample of all men diagnosed with invasive prostate cancer (ICD10 C61) between 1st January 1995 and 31st March 2010, and alive in November 2011, was selected from the two population-based cancer registries in Ireland (*n* = 22,823). From this, a country-stratified random sample of 12,322 men was selected. Healthcare professionals screened men for eligibility to participate. Men were eligible if they were (i) alive, (ii) aware of their diagnosis, (iii) well enough to complete a questionnaire, (iv) able to understand English and (v) resident in RoI or NI. Six thousand five hundred fifty-nine survivors, who were between 2 and 18 years post-diagnosis, were deemed eligible to receive a questionnaire. Questionnaires were posted during April to September 2012. Non-responders received up to two written reminders and free-phone numbers were provided if help was required.

### Ethical approval

All procedures were in accordance with the ethical standards of the Irish College of General Practitioners and the Office for Research Ethics Committee NI and with the 1964 Helsinki Declaration and its later amendments [17]. Research governance approval was obtained from the five NI Health Trusts. Informed consent was obtained from all individual participants included in the study through return of completed questionnaires and/or consent forms.

### Survey instrument

Men were asked how their prostate cancer was detected. They were classified as having asymptomatic ‘PSA-detected’ disease if they answered ‘yes’ to any of the following statements: ‘I had no symptoms and my GP offered to test my PSA as part of a general health check’ or ‘I had no symptoms and I asked my GP to measure my PSA’, and as having symptomatic ‘clinically detected’ disease if they answered ‘yes’ to either of the following statements: ‘I attended my GP with urinary symptoms (e.g. urinating frequently, blood in urine)’ or ‘I attended my GP with other symptoms (e.g. back pain, joint pain)’. Respondents who provided free text descriptions were categorised as asymptomatic PSA detected or symptomatic clinically detected as appropriate. Men who endorsed both asymptomatic and symptomatic statements (*n* = 171; 5 % all respondents) were coded as symptomatic. Hereafter these groups are referred to as PSA detected and clinically detected.

The questionnaire asked about treatment(s) received with start and end dates, adverse physical effects experienced ‘ever’ after treatment and ‘ongoing’ at questionnaire completion; comorbid conditions at diagnosis and whether they were treated for depression following their prostate cancer diagnosis. Content also included socio-demographic characteristics.

Psychological wellbeing, in the week prior to questionnaire completion, was assessed using the 21 question version of the Depression Anxiety Stress Scales (DASS-21). DASS-21 includes three sub-scales relating to self-reported negative emotional states: depression, anxiety and stress [18]. Each subscale comprises seven questions. Respondents were asked to respond to each question along a 4-point Likert scale from 0 ‘Did not apply to me at all’ to 3 ‘Applied to me very much, or most of the time’. The depression scale assesses dysphoria, hopelessness, devaluation of life, self-deprecation, lack of interest/involvement, anhedonia and inertia. The anxiety scale assesses autonomic arousal, skeletal muscle effects, situational anxiety and subjective experience of anxious affect, and the stress scale assesses levels of chronic non-specific arousal, difficulty relaxing, nervous arousal and being easily upset/agitated, irritable/over-reactive and impatient [18]. The Cronbach’s alpha value for the 7-item scales of DASS-21 ranges from 0.73 (anxiety) to 0.81 (stress and depression), and it has adequate convergent and discriminate validity [18.19].

### Data handling

Scores for each DASS-21 subscale were calculated by summing scores for the relevant questions [18]. A maximum score of 21 could be achieved for each subscale and scores were doubled for analysis. Depression, anxiety and stress were analysed separately, with scores reduced to binomial variables for modelling. Respondents were categorised as having depression, anxiety or stress if they scored ≥10, ≥8 or ≥15 on the relevant subscale [18]. Men who completed all questions on a sub-scale were included in analysis of that sub-scale.

A mutually exclusive primary treatment variable was constructed with categories radical prostatectomy (RP) with or without other treatments, external beam radiotherapy (EBRT) with concurrent androgen deprivation therapy (ADT) but without RP, EBRT without concurrent ADT or RP, brachytherapy (BT) without RP or EBRT, ADT only or active surveillance (AS)/watchful waiting (WW). Five men received chemotherapy (CT) only as primary treatment and 93 did not respond to treatment questions; these 98 men were excluded from analyses. Men who received ADT at questionnaire completion were defined as ‘current ADT’. Responders were classified as ‘CT treated’ if they ever received CT. All participants had survived at least 2 years post-diagnosis; therefore, those currently receiving ADT [20] and treated with CT were considered as having advanced/recurrent disease. Survivors were grouped according to length of time since diagnosis: <5 years, 5–9.9 years and ≥10 years post-diagnosis, corresponding to short, long and long-term survivors, respectively.

Questionnaire data were linked with cancer registration data to obtain date of diagnosis, clinical stage at diagnosis (Tumour-Lymph Node-Metastasis (TNM) classification, version 5) and Gleason grade (GG) at diagnosis (low 2–4, medium 5–7, high 8–10).

### Statistical analysis

Analyses were performed using STATA v13.1 (StataCorp LP, 2013). Where more than 5 % of respondents declined to answer a question, a ‘not reported’ category was included. Prevalence of depression, anxiety and stress was compared by mode of detection (PSA tested/clinically detected) using *t* tests. Chi-square tests were used to investigate associations between mode of detection and socio-demographic and clinical characteristics. Prevalence of depression, anxiety and stress by time since diagnosis was also investigated by chi-square tests.

The primary outcome variables were depression, anxiety and stress. A three-stage process was used for model fitting. Firstly, a univariate odds ratio (OR) was computed for association between mode of detection and each psychological outcome. Socio-demographic and clinical characteristics likely differ between those with PSA-detected and clinically detected prostate cancer [21]; therefore, clinical (GG, stage and comorbidities at diagnosis, family history of prostate cancer and treated for depression) and socio-demographic (age at diagnosis, marital status, living alone, educational attainment, employment status at questionnaire completion, jurisdiction and time since diagnosis) factors were considered as potential confounders of associations between mode of detection and psychological wellbeing.

In the second stage, socio-demographic and clinical variables significant at the 5 % level in univariate analyses were considered for inclusion in models for depression, anxiety and stress. Variables significant at the 5 % level were retained. These risk estimates were termed ‘adjusted ORs (AOR)’.

Primary treatments and adverse physical effects experienced may also differ between men with PSA-detected and clinically detected cancer. Therefore, in the third stage, the AOR for mode of detection was further adjusted by individually fitting primary treatment(s) received, current ADT, CT treated and each ongoing adverse physical effect, to the adjusted models for depression, anxiety and stress. These variables were fitted individually because of concerns regarding collinearity and the potential for over-adjustment. These risk estimates were termed ‘multivariate ORs (MVOR)’.

Sensitivity analyses were conducted where all men who (i) completed questionnaires by telephone (*n* = 60), (ii) were treated for depression post-diagnosis (*n* = 167) and (iii) had evidence of advanced/recurrent disease (*n* = 721) were removed, and adjusted and multivariate models were rerun.

## Results

The response rate was 54 % (3348/6262) when men discovered to be ineligible following questionnaire dispatch (e.g. recent death, incorrect address; *n* = 297) were removed from the denominator. Men from RoI and those who were ≤59 years at diagnosis were significantly more likely to respond than older men and those from NI (Supplementary Table 1) [16]. Significantly more respondents than non-respondents were staged and graded (*p* < 0.001). No significant difference in response was observed by time since diagnosis.

Of all respondents, 1978 (59 %) had PSA-detected and 1331 (40 %) had clinically detected prostate cancer (Table 1). This information was missing for 1 % of respondents (*n* = 39).

**Table 1:** Characteristics of the respondents overall and grouped by mode of detection (asymptomatic PSA-tested and symptomatic clinically detected prostate cancer) with chi-squared *p* values

		All respondents	PSA detected/asymptomatic^a^	Clinically detected/symptomatic^a^
		*N* = 3348	*N* = 1978	*N* = 1331
		*N* (%)	*N* (%)	*N* (%)
Jurisdiction	RoI	2338 (70 %)	1538 (78 %)	766 (58 %)
	NI	1010 (30 %)	440 (22 %)	565 (42 %)***
Age at diagnosis (years)	≤59	799 (24 %)	514 (26 %)	276 (21 %)
	60–69	1631 (49 %)	953 (48 %)	662 (50 %)
	≥70	918 (27 %)	511 (26 %)	393 (29 %)***
Time since diagnosis	<5 years	1614 (48 %)	990 (50 %)	606 (46 %)
	5–9.9 years	1075 (32 %)	656 (33 %)	407 (31 %)
	≥10 years	659 (20 %)	332 (17 %)	318 (24 %)***
Marital status	Married/cohabiting	2753 (82 %)	1638 (83 %)	1098 (83 %)
	Not married	558 (17 %)	328 (17 %)	223 (17 %)
	Not reported	37 (1 %)	12 (0.6 %)	10 (1.0 %)
Highest educational level attained	Primary	1187 (36 %)	618 (31 %)	557 (42 %)
	Secondary	1122 (34 %)	729 (37 %)	387 (29 %)
	Tertiary	899 (27 %)	562 (28 %)	334 (25 %)
	Not reported	140 (4 %)	69 (4 %)	53 (4 %)***
Employment status^b^	Employed	1124 (34 %)	715 (36 %)	399 (30 %)
	Retired	336 (12 %)	228 (12 %)	154 (12 %)
	NA^d^	1802 (54 %)	1014 (51 %)	765 (58 %)
	Not reported	36 (1 %)	21 (1 %)	13 (1.0 %)**
Family history of prostate cancer	Yes	791 (24 %)	500 (26 %)	288 (22 %)
	No	2448 (73 %)	1434 (74 %)	998 (78 %)
	Not reported	109 (3 %)	44 (2 %)	45 (3 %)*
Comorbidity at diagnosis	None	1476 (44 %)	941 (48 %)	513 (39 %)
	Any	1872 (56 %)	1037 (52 %)	818 (62 %)***
Treated for depression^c^	Yes	176 (5 %)	78 (4 %)	89 (7 %)
	No	3181 (95 %)	1900 (96 %)	1242 (93 %)***
Gleason grade at diagnosis	Low (≤6)	212 (6 %)	116 (6 %)	90 (7 %)
	Medium (7–8)	2186 (65 %)	1356 (69 %)	808 (61 %)
	High (8–10)	625 (19 %)	332 (17 %)	288 (22 %)
	Unknown	325 (10 %)	174 (9 %)	145 (11 %)***
Clinical stage at diagnosis	I	18 (1 %)	8 (0.4 %)	10 (1.0 %)
	II	1875 (56 %)	1254 (63 %)	599 (45 %)
	III	463 (14 %)	233 (12 %)	226 (17 %)
	IV	141 (4 %)	51 (3 %)	88 (7 %)
	Unknown	851 (25 %)	432 (22 %)	408 (31 %)***
Primary treatment	RP	934 (28 %)	607 (31 %)	316 (25 %)
	EBRT with concurrent ADT	591 (18 %)	358 (19 %)	232 (18 %)
	EBRT without concurrent ADT	1127 (34 %)	652 (34 %)	463 (36 %)
	BT	124 (4 %)	104 (5 %)	20 (2 %)
	ADT	310 (9 %)	137 (7 %)	167 (13 %)
	AS/WW	164 (5 %)	82 (4 %)	79 (6 %)***
Current ADT^b^	Yes	657 (20 %)	317 (16 %)	338 (25 %)
	No/not reported	2691 (80 %)	1661 (84 %)	993 (75 %)***
CT treated	Yes	64 (2 %)	25 (1 %)	38 (3 %)
	No/not reported	3284 (98 %)	1953 (99 %)	1293 (97 %)**
Incontinence^b^	Ongoing	538 (16 %)	274 (14 %)	258 (19 %)
	No/not reported	2810 (84 %)	1704 (86 %)	1073 (81 %)***
Impotence^b^	Ongoing	1960 (59 %)	1207 (61 %)	739 (56 %)
	No/not reported	1388 (41 %)	771 (39 %)	592 (44 %)**
Loss of libido^b^	Ongoing	1572 (47 %)	917 (46 %)	646 (49 %)
	No/not reported	1776 (53 %)	1061 (54 %)	685 (51 %)
Bowel problems^b^	Ongoing	496 (15 %)	247 (13 %)	244 (18 %)
	No/not reported	2852 (85 %)	1731 (87 %)	1087 (82 %)***
Gynecomastia^b^	Ongoing	350 (10 %)	181 (9 %)	167 (13 %)
	No/not reported	2998 (90 %)	1797 (91 %)	1164 (87 %)**
Hot flashes/sweats^b^	Ongoing	582 (17 %)	274 (14 %)	305 (23 %)
	No/not reported	2766 (83 %)	1704 (86 %)	1026 (77 %)***
Fatigue^b^	Ongoing	765 (23 %)	376 (19 %)	385 (29 %)
	No/not reported	2583 (77 %)	1602 (81 %)	946 (71 %)***

*RoI* Republic of Ireland, *NI* Northern Ireland; primary treatment hierarchy: *RP* radical prostatectomy, *EBRT* external beam radiotherapy, *ADT* androgen deprivation therapy, *BT* brachytherapy, *AS/WW* active surveillance/watchful waiting, *CT* chemotherapy

*p < 0.05; **p < 0.01; ***p < 0.001

^**a**^Men were classified as having asymptomatic ‘PSA-detected’ disease if they answered ‘yes’ to any of the following statements: ‘I had no symptoms and my GP offered to test my PSA as part of a general health check’ or ‘I had no symptoms and I asked my GP to measure my PSA’, and as having symptomatic ‘clinically detected’ disease if they answered ‘yes’ to either of the following statements: ‘I attended my GP with urinary symptoms (e.g. urinating frequently, blood in urine)’ or ‘I attended my GP with other symptoms (e.g. back pain, joint pain)’. Men who endorsed both asymptomatic and symptomatic statements (*n* = 171; 5 %) were coded as symptomatic

^**b**^At the time of questionnaire completion

^**c**^Treated for depression post-prostate cancer diagnosis

^d^Not working at the time of diagnosis or at questionnaire completion

The percentage of men with PSA-detected prostate cancer was significantly higher among men who were from RoI, younger at diagnosis, employed at questionnaire completion and <10 years post-diagnosis and had secondary- or third-level education, a family history of prostate cancer and without comorbidities at diagnosis (Table 1). PSA-detected men were significantly more often diagnosed with stage II and medium GG cancers than clinically detected men. Primary treatment(s) received varied significantly with mode of detection; a higher percentage of PSA-detected men received RP or BT, while a higher percentage of clinically detected men were treated with ADT alone. The percentage of men currently receiving ADT, or treated with CT, was significantly higher among men with PSA-detected than clinically detected disease. Clinically detected men significantly more often reported ongoing urinary incontinence, hot flashes, bowel problems, gynecomastia and fatigue than survivors with PSA-detected disease; only impotence was significantly higher among men diagnosed with PSA-detected prostate cancer. Loss of libido did not differ significantly by mode of diagnosis.

**Fig. 1:**
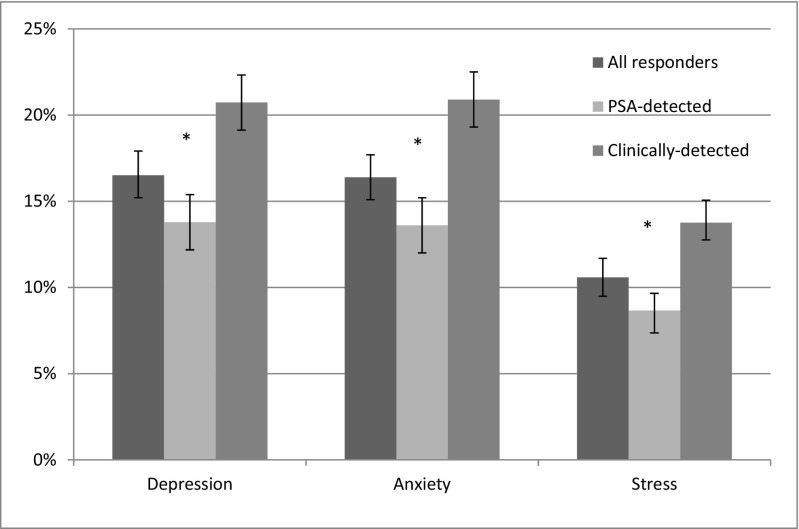
Prevalence of depression, anxiety and stress overall among prostate cancer survivors, and by mode of detection (PSA-detected and clinically detected prostate cancer), with 95 % confidence intervals

Overall, prevalence of depression, anxiety and stress among respondents was 17 % (95 % CI 15.2 %, 17.9 %), 16 % (95 % CI 15.1 %, 17.7 %) and 11 % (95 % CI 9.5 %, 11.7 %), respectively (Fig. 1). Prevalence of each negative emotional state did not vary significantly with time since diagnosis (depression *p* = 0.958; anxiety *p* = 0.380 and stress; *p* = 0.461).

Survivors with PSA-detected disease had significantly lower prevalence of depression (14 vs 21 %; *p* < 0.001), anxiety (14 vs 21 %; *p* < 0.001) and stress (9 vs 14 %; *p* < 0.001) than those with clinically detected disease (Fig. 1). Clinically detected survivors were significantly more likely to have been treated for depression post-prostate cancer diagnosis than PSA-detected survivors (7 vs 4 %; *p* = 0.001)..

In univariate analysis, mode of detection was significantly associated with each negative psychological state; survivors with clinically detected disease had significantly higher risks of depression, anxiety and stress than those with PSA-detected disease (Table 2).

**Table 2:** Significant associations between mode of detection (PSA-detected and clinically detected prostate cancer) and socio-demographic and clinical variables and depression, anxiety and stress; univariate and adjusted odds ratios (OR) with 95 % confidence intervals

		Depression		Anxiety		Stress	
		Univariate OR (95 % CI)	Adjusted OR^a^ (95 % CI)	Univariate OR (95 % CI)	Adjusted OR^a^ (95 % CI)	Univariate OR (95 % CI)	Adjusted OR^a^ (95 % CI)
Mode of detection^b^	PSA detected	1	1	1	1	1	1
	Clinically detected	1.64 (1.34, 1.99)***	1.46 (1.18, 1.80) **	1.68 (1.38, 2.04)***	1.36 (1.09, 1.68) **	1.68 (1.33, 2.13)***	1.43 (1.11, 1.85) *
Socio-demographic factors							
Age at diagnosis (years)	≤59	1	1			1	1
	60–69	0.70 (0.56, 0.89)	0.59 (0.45, 0.78)			0.74 (0.56, 0.98)	0.69 (0.51, 0.93)
	≥70	0.76 (0.58, 0.99)*	0.54 (0.39, 0.75)***			0.64 (0.46, 0.89)*	0.57 (0.39, 0.81)*
Marital status	Married	1	1	1	1		
	Other	1.42 (1.11, 1.82)*	1.39 (1.06, 1.81)*	1.51 (1.18, 1.92)**	1.43 (1.10, 1.86)*		
Living alone	No	1		1			
	Yes	1.45 (1.10, 1.91)*		1.62 (1.24, 2.12)**			
Jurisdiction	RoI	1		1	1	1	
	NI	1.38 (1.12, 1.69)*		1.46 (1.19, 1.79)***	1.27 (1.01, 1.59)*	1.45 (1.14, 1.84)*	
Highest educational level attained	Primary	1	1	1	1	1	1
	Secondary	0.72 (0.58, 0.91)	0.79 (0.61, 1.01)	0.64 (0.51, 0.81)	0.76 (0.59, 0.96)	0.65 (0.49, 0.86)	0.69 (0.51, 0.93)
	Tertiary	0.60 (0.46, 0.76)***	0.60 (0.46, 0.79)***	0.51 (0.40, 0.66)***	0.54 (0.41, 0.71)***	0.55 (0.40, 0.75)***	0.53 (0.39, 0.74)***
Employment status at questionnaire completion	Employed	1	1	1		1	
	Not employed	1.59 (1.15, 2.19)	1.29 (0.91, 1.84)	1.47 (1.05, 2.05)		1.63 (1.10, 2.40)	
	NA	1.45 (1.16, 1.82)***	1.50 (1.15, 1.97)*	1.60 (1.28, 2.00)***		1.45 (1.11, 1.90)*	
Clinical factors							
Comorbidity at diagnosis	None	1	1	1	1	1	1
	Any	1.89 (1.54, 2.32)***	1.63 (1.31, 2.04)***	2.35 (1.89, 2.90)***	2.07 (1.65, 2.59)***	2.14 (1.66, 2.77)***	1.99 (1.51, 2.62)***
Treated for depression	Yes	1	1	1	1	1	1
	No	7.38 (5.23, 10.43)***	5.90 (4.08, 8.51)***	7.21 (5.11, 10.18)***	6.49 (4.51, 9.33)***	9.27 (6.52, 13.18)***	7.64 (5.28, 11.06)***

*RoI* Republic of Ireland, *NI* Northern Ireland, *NA* neither employed at diagnosis or questionnaire completion

*p < 0.05; **p < 0.01; ***p < 0.001

^**a**^Adjusted ORs are mutually adjusted for variables shown in the columns; likelihood ratio *p* values for adjusted ORs are shown

^**b**^Men were classified as having asymptomatic ‘PSA-detected’ disease if they answered ‘yes’ to any of the following statements: ‘I had no symptoms and my GP offered to test my PSA as part of a general health check’ or ‘I had no symptoms and I asked my GP to measure my PSA’, and as having symptomatic ‘clinically detected’ disease if they answered ‘yes’ to either of the following statements: ‘I attended my GP with urinary symptoms (e.g. urinating frequently, blood in urine)’ or ‘I attended my GP with other symptoms (e.g. back pain, joint pain)’. Men who endorsed both asymptomatic and symptomatic statements (*n* = 171; 5 %) were coded as symptomatic

Risk of depression, anxiety and stress was highest among survivors who were unmarried, unemployed and from NI; had primary-level education and ≥1 comorbidity at diagnosis; and were treated for depression post-diagnosis. Survivors ≤59 years at diagnosis had higher odds of depression and stress than older men. Odds of depression and anxiety were significantly higher in those who lived alone, compared to those who did not.

In adjusted models containing significant socio-demographic and clinical factors, mode of diagnosis remained significantly associated with psychological wellbeing; clinically detected men had higher odds of depression (AOR = 1.46, 95 % CI 1.18, 1.80), anxiety (AOR = 1.36, 95 % CI 1.09, 1.68) and stress (AOR = 1.43, 95 % CI 1.11, 1.85) than those with PSA-detected disease (Table 2).

Primary treatment(s) (*p* = 0.047) and having received CT (*p* = 0.035) were significantly associated with increased odds of anxiety, and current ADT was associated with increased odds of anxiety (*p* = 0.015) and stress (*p* < 0.001). Risk of each negative psychological state was significantly higher in men who experienced each ongoing adverse physical effect than those who did not, and risk increased significantly with number of adverse physical effects experienced (all *p* values <0.05; data not shown).

When each treatment and adverse physical effect variable was added individually to adjusted models, mode of detection remained significantly associated with depression (range of MVORs 1.35 to 1.49) and anxiety (range of MVORs 1.27 to 1.38; table 3). Mode of detection also remained significantly associated with stress when each treatment and adverse effect variable was added to the adjusted model (range of MVORs 1.36 to 1.44), except for fatigue and total number of adverse effects.

**Table 3:** Multivariate odds ratios (MVOR) with 95 % confidence intervals and likelihood ratio *p* values for mode of detection (PSA detected and clinically detected). MVORs for mode of detection are adjusted for significant socio-demographic and clinical factors (Table 2) and individually for treatment and adverse physical effects variables

	Mode of detection^a^	Depression	Anxiety	Stress
		Multivariate OR (95 % CI)	Multivariate OR (95 % CI)	Multivariate OR (95 % CI)
Prostate cancer treatments				
Primary treatment(s)^b^	PSA detected	1	1	1
	Clinically detected	1.44 (1.16, 1.79)**	1.31 (1.05, 1.63)*	1.41 (1.09, 1.82)*
Current ADT	PSA detected	1	1	1
	Clinically detected	1.44 (1.16, 1.80)**	1.33 (1.07, 1.65)*	1.39 (1.07, 1.80)*
Treated with CT	PSA detected	1	1	1
	Clinically detected	1.45 (1.18, 1.80)**	1.33 (1.07, 1.66)*	1.42 (1.10, 1.83)*
Adverse physical effects ongoing at questionnaire completion				
Incontinence	PSA detected	1	1	1
	Clinically detected	1.40 (1.13, 1.73)*	1.27 (1.02, 1.58)*	1.36 (1.05, 1.76)*
Impotence	PSA detected	1	1	1
	Clinically detected	1.49 (1.20, 1.84)**	1.38 (1.11, 1.72)*	1.44 (1.12, 1.87)*
Loss of libido	PSA detected	1	1	1
	Clinically detected	1.46 (1.18, 1.80)**	1.37 (1.10, 1.70)*	1.42 (1.10, 1.84)**
Bowel problems	PSA detected	1	1	1
	Clinically detected	1.42 (1.15, 1.76)**	1.33 (1.07, 1.65)*	1.37 (1.06, 1.77)*
Gynacomastia	PSA detected	1	1	1
	Clinically detected	1.44 (1.17, 1.78)**	1.35 (1.09, 1.67)*	1.41 (1.09, 1.83)*
Hot flashes/sweats	PSA detected	1	1	1
	Clinically detected	1.43 (1.15, 1.76)**	1.35 (1.09, 1.67)*	1.36 (1.05, 1.76)*
Fatigue	PSA detected	1	1	1
	Clinically detected	1.35 (1.09, 1.68)**	1.28 (1.03, 1.60)*	1.29 (0.99, 1.67)
Total number adverse physical effects	PSA detected	1	1	1
	Clinically detected	1.35 (1.09, 1.68)*	1.28 (1.03, 1.60)*	1.27 (0.98, 1.65)

*p < 0.05; **p < 0.01; ***p < 0.001

^**a**^Men were classified as having asymptomatic ‘PSA-detected’ disease if they answered ‘yes’ to any of the following statements: ‘I had no symptoms and my GP offered to test my PSA as part of a general health check’ or ‘I had no symptoms and I asked my GP to measure my PSA’, and as having symptomatic ‘clinically detected’ disease if they answered ‘yes’ to either of the following statements: ‘I attended my GP with urinary symptoms (e.g. urinating frequently, blood in urine)’ or ‘I attended my GP with other symptoms (e.g. back pain, joint pain)’. Men who endorsed both asymptomatic and symptomatic statements (*n* = 171; 5 %) were coded as symptomatic

^**b**^Primary treatment: a hierarchical variable defined as RP, radical prostatectomy with/without other treatments; ERBT, external beam radiotherapy; BT, brachytherapy; ADT, androgen deprivation therapy; AS/WW, active surveillance/watchful waiting; and CT, chemotherapy

Mode of detection remained significant in both adjusted and multivariate models of depression, anxiety and stress in each of the three sensitivity analyses (data not shown).

## Discussion

To inform rational decision-making around PSA testing, it is essential to have a comprehensive understanding of consequent benefits and risks. To extend understanding in this area, we investigated—for the first time to our knowledge—whether long-term psychological wellbeing differed between prostate cancer survivors detected through PSA testing and those who were not. In our large, population-based study of men 2 to 18 years post-diagnosis, we found that those diagnosed with asymptomatic PSA-detected prostate cancer had significantly reduced odds of depression, anxiety and stress compared to men with symptomatic clinically detected disease, after controlling for socio-demographic and clinical factors.

In this study, men with PSA-detected prostate cancer were younger and had lower-stage and lower-grade disease and fewer comorbidities at diagnosis than those with clinically detected disease. This is similar to characteristics of screened populations in randomised controlled screening trials [22, 23]. However, we adjusted associations between mode of detection and psychological wellbeing for these variables; therefore, they cannot explain our findings.

Screening can lead to less aggressive treatment for early screen-detected cancers, e.g. breast cancer [24], but this is not the case for PSA-detected prostate cancer. Indeed, only 5 % of survivors in this dataset were managed by active surveillance/watchful waiting. Different primary treatment patterns were observed between men with PSA-detected and clinically detected disease, and primary treatment was significantly associated with increased risk of anxiety (but not depression or stress), following adjustment for socio-demographic and clinical factors. There is limited and inconsistent information regarding treatment effects on prostate cancer survivors’ psychological health [25–28]. Men currently receiving ADT had significantly increased odds of anxiety and stress, and anxiety risk was increased in those treated with CT. These men are likely to have recurrences/disease progression [20], which increases risk of poor psychological health among men with prostate cancer [29]. When we adjusted for treatments, mode of diagnosis remained significantly associated with increased risk of depression, anxiety and stress, so differences in treatment between PSA-detected and clinically detected men cannot explain our results.

Risk of all three negative psychological states was significantly higher in survivors who experienced each ongoing adverse physical effect, compared to those who did not. Furthermore, risk of depression, anxiety and stress increased with the number of adverse physical effects. This finding is in agreement with previous studies which found that loss of previous abilities post-treatment increased odds of poor psychological wellbeing among prostate cancer survivors [26, 30]. Indeed, loss of quality-adjusted life years due to long-term adverse effects of prostate cancer decreased the benefit of PSA screening in the ERSPC trial [22]. Within this dataset, survivors with PSA-detected disease less often reported ongoing adverse physical effects, except for impotence, than men with clinically detected disease. Furthermore, men with PSA-detected cancer were also at lower risk of poor psychological health when models were adjusted for each adverse physical effect, or number of adverse effects, than those with clinically detected disease.

So how might we explain our findings? Firstly, results may be explained by (self) selection, i.e. systematic differences in men who have PSA tests and those who do not. The ‘healthy user effect’, i.e. the propensity of healthier patients to seek out and initiate preventative therapies, is one possible explanation. For example, Brookhart et al. [31] found that people who were adherent to statin therapy were more likely to have cancer screening tests, including PSA tests, than those who were not adherent. This they hypothesise may be due to (i) adherent patients being ‘more health seeking’ and therefore more likely to seek or agree to take other preventative interventions and tests and/or (ii) differences in health status (physical and cognitive) between the two groups, with those who are adherent being healthier. Additionally, men with poorer psychological health have been found to be less likely to have PSA tests [32–34]. Moreover, those predisposed to poorer psychological health are also predisposed to poor wellbeing throughout their disease trajectory [35]. Therefore, despite adjusting for a range of socio-demographic and clinical variables known to be associated with likelihood of PSA testing, it is possible that our findings are due to unmeasured confounders between men with PSA-detected and clinically detected prostate cancer, for example their psychosocial functioning and their relationships.

Another possibility is that those diagnosed with PSA-detected and clinically detected disease adjusted to their ‘new normal’ differently. Men diagnosed with PSA-detected prostate cancer, because they were asymptomatic, may consider themselves ‘lucky’ their cancer was discovered. They may also attribute their survival to treatment received and be grateful for being cured [36]. We speculate that such feelings of good fortune could translate into better psychological wellbeing.

Many issues have been shown to be associated with this adjustment including self-efficacy [37], perceived stress [37], dyadic adjustment and threat appraisal [38]. Furthermore, a person’s appraisal of the significance of an event or stress, e.g. a cancer diagnosis, is influenced by their characteristics and their environment; this in turn influences their subsequent coping style [39]. Coping style, i.e. the thoughts and behaviours used to regulate distress, is central to the adjustment process [37–40]. Men with prostate cancer use different coping strategies depending on age, PSA level and stage [40, 41]. Younger men, with lower PSA levels were more likely to use positive coping skills including problem-solving and self-reliance, which in turn were associated with lower levels of depression [40]. More negative coping skills, including cognitive avoidance, have been shown to be used by men with later-stage disease, and these predict anxiety [41]. Therefore, coping strategies may differ between men with PSA-detected and clinically detected prostate cancer in such a way as to result in better psychological wellbeing in the former group. Finally, adequate communication between men with prostate cancer, their partners and their medical teams is also important in adjustment [42], and those requesting and/or accepting PSA tests may be most likely to engage with their doctors and/or partners.

Overall, however, these explanations are largely speculative and further research, including longitudinal investigations of the evolution of negative psychological states, and associated factors, following men diagnosed with symptomatic, clinically detected and asymptomatic PSA-detected prostate cancer from diagnosis through the survivorship continuum are needed. Furthermore, we support the recommendations by the National Comprehensive Cancer Network and others that patients and survivors with (prostate) cancer should be screened regularly for psychological distress, ‘the sixth vital sign’, and referred for appropriate supportive care. Interventions have been shown to reduce or alleviate negative psychological symptoms among men with prostate cancer [43]; however, in light of our findings, we suggest that the effect of these supportive care interventions on men diagnosed with clinically detected and PSA-detected prostate cancer should be investigated.

Prevalence of depression and anxiety among survivors did not vary significantly with time since diagnosis, suggesting that it may be persistent for some men post-treatment. Prevalence of depression and anxiety in this dataset were similar, although slightly lower than that previously reported (depression 18.4 %, anxiety 18.5 % [28]. Comparisons between studies are difficult due to variations in psychological assessment tools used and socio-demographic, clinical and treatment characteristics of datasets. Notwithstanding this, prevalence of depression among prostate cancer survivors overall was higher than the 10 % reported in the Irish population ≥50 years [44], and among survivors with clinically detected prostate cancer, prevalence of depression (21 vs 18.3 %) and anxiety (21 vs 5.6 %) was higher than that among a UK adult population [19].

This population-based study extends current knowledge about the psychological health of prostate cancer survivors; a meta-analysis of 27 studies across all phases of the disease trajectory included 3087 men post-treatment; [28] this study alone involved 3348 survivors post-treatment. It is the first to investigate the effect of mode of detection on prostate cancer survivors’ psychological wellbeing. It is the first time all common treatments have been directly compared for their effect on the psychological health of survivors. Furthermore, we used a validated instrument to investigate psychological wellbeing [18]. However, the study was cross-sectional and measured depression, anxiety and stress in the week prior to questionnaire completion; therefore, we do not know how many men become depressed, anxious or stressed at different stages during survivorship and who had recovered by the time of questionnaire completion. We did not have information on the pre-diagnostic history of, or predisposition to, depression, anxiety or stress. Missing data in DASS-21 means that prevalence of depression, anxiety and/or stress may have been over- or underestimated, and non-responders may have differed in the effect of mode of detection on their psychological wellbeing than responders. Finally, we based our analysis on self-reported treatment(s) and mode of detection and, like all patient-reported outcomes research, there is a possibility of recall bias within our study [45].

In this study, more than one in six prostate cancer survivors experienced poor psychological health and risk was significantly higher in men with clinically detected disease. PSA testing is common in most developed countries, and some advocate the implementation of prostate cancer screening programmes [2–4]. To inform rational decision-making, it is imperative that all potential benefits and risks of testing are assessed; this study fills a gap in the evidence base around PSA testing. Irrespective of PSA testing policy, the relatively high prevalence of negative psychological states among survivors in this study means that a ‘risk-adapted approach’ should be implemented to screen survivors most at risk of psychological morbidity for psychological health. Our findings suggest that mode of detection could be considered as a risk stratum in such an approach. This, together with increased utilisation of medications and/or cognitive interventions to improve psychological wellbeing, may enhance clinical outcomes and improve psychological wellbeing among prostate cancer survivors.

In conclusion, prostate cancer survivors diagnosed with asymptomatic PSA-detected disease were at lower risk of depression, anxiety and stress during survivorship than those with symptomatic clinically diagnosed disease, following adjustment for socio-demographic and clinical factors. Our findings require further research to understand the underlying reasons. These findings contribute to the ongoing debate on the benefits and risks of PSA testing and may be considered by policy makers formulating population-based prostate cancer screening policies.

## Electronic supplementary material

ESM 1 (DOCX 15 kb)

## References

[1] Raffle AE, Gray JAM (2007) Screening: evidence and practice. Oxford University Press

[2] McCarthy M (2014). Canadian panel recommends against PSA screening. BMJ.

[3] Rychetnik L, Doust J, Thomas R, Gardiner R, Mackenzie G, Glasziou P (2014). A community jury on PSA screening: what do well-informed men want the government to do about prostate cancer screening–a qualitative analysis. BMJ Open.

[4] Drummond FJ, Carsin AE, Sharp L, Comber H (2010). Trends in prostate specific antigen testing in Ireland: lessons from a country without guidelines. Ir J Med Sci.

[5] Potosky AL, Davis WW, Hoffman RM, Stanford JL, Stephenson RA, Penson DF, Harlan LC (2004). Five-year outcomes after prostatectomy or radiotherapy for prostate cancer: the prostate cancer outcomes study. J Natl Cancer Inst.

[6] Carsin AE, Drummond FJ, Black A, van Leeuwen PJ, Sharp L, Murray LJ, Connolly D, Egevad L, Boniol M, Autier P, Comber H, Gavin A (2010). Impact of PSA testing and prostatic biopsy on cancer incidence and mortality: comparative study between the Republic of Ireland and Northern Ireland. Cancer Causes Control.

[7] Schröder FH, Hugosson J, Roobol MJ, Tammela TL, Zappa M, Nelen V, Kwiatkowski M, Lujan M, Määttänen L, Lilja H, Denis LJ, Recker F, Paez A, Bangma CH, Carlsson S, Puliti D, Villers A, Rebillard X, Hakama M, Stenman UH, Kujala P, Taari K, Aus G, Huber A, van der Kwast TH, van Schaik RH, de Koning HJ, Moss SM, Auvinen A, Investigators ERSPC (2014). Screening and prostate cancer mortality: results of the European Randomised Study of Screening for Prostate Cancer (ERSPC) at 13 years of follow-up. Lancet.

[8] Andriole GL, Crawford ED, Grubb RL, Buys SS, Chia D, Church TR, Fouad MN, Isaacs C, Kvale PA, Reding DJ, Weissfeld JL, Yokochi LA, O’Brien B, Ragard LR, Clapp JD, Rathmell JM, Riley TL, Hsing AW, Izmirlian G, Pinsky PF, Kramer BS, Miller AB, Gohagan JK, Prorok PC, Project Team PLCO (2012). Prostate cancer screening in the randomized Prostate, Lung, Colorectal, and Ovarian Cancer Screening Trial: mortality results after 13 years of follow-up. J Natl Cancer Inst.

[9] Ilic D, Neuberger MM, Djulbegovic M, Dahm P (2013). Screening for prostate cancer. Cochrane Database Syst Rev.

[10] Katz DA, Jarrard DF, McHorney CA, Hillis SL, Wiebe DA, Fryback DG (2007). Health perceptions in patients who undergo screening and workup for prostate cancer. Urology.

[11] Scott JG, Shaw EK, Friedman A (2013). Emotional consequences of persistently elevated PSA with negative prostate biopsies. Am J Can Prev.

[12] Mitchell AJ, Ferguson DW, Gill J, Paul J, Symonds P (2013). Depression and anxiety in long-term cancer survivors compared with spouses and healthy controls: a systematic review and meta-analysis. Lancet Oncol.

[13] Yang YL, Liu L, Wang Y, Wu H, Yang XS, Wang JN, Wang L (2013). The prevalence of depression and anxiety among Chinese adults with cancer: a systematic review and meta-analysis. BMC Cancer.

[14] National Prostate Cancer General Practitioner Referral Guidelines. National Cancer Control Programme. http://www.healthlink.ie/Oncology/NCCP%20Prostate%20Cancer%20Referral%20Guideline%20Version%201.3%20January%202011.pdf. Accessed 8th December 2011

[15] Prostate Cancer: diagnosis and treatment. National Institute for Health and Care Excellence (NICE). http://www.nice.org.uk/guidance/cg175/chapter/1-recommendations, Accessed 9th July 2014

[16] Drummond FJ, Kinnear H, Donnelly C, O’Leary E, O’Brien K, Burns RM, Gavin A, Sharp L (2015). Establishing a population-based patient-reported outcomes study (PROMs) using national cancer registries across two jurisdictions: the Prostate Cancer Treatment, your experience (PiCTure) study. BMJ Open.

[17] WMA Declaration of Helsinki—ethical principles for medical research involving human subjects (2008). http://www.wma.net/en/30publications/10policies/b3/17c.pdf. Accessed 9 July 2014.

[18] Lovibond SH, Lovibond PF (1995). Manual for the Depression Anxiety Stress Scales 2nd. Ed.

[19] Crawford JR, Henry JD (2003). The Depression Anxiety Stress Scales (DASS): normative data and latent structure in a large non-clinical sample. Br J Clin Psychol.

[20] Glaser AW, Fraser LK, Corner J, Feltbower R, Morris EJ, Hartwell G, Richards M, Wagland R (2013). Patient-reported outcomes of cancer survivors in England 1-5 years after diagnosis: a cross-sectional survey. BMJ Open.

[21] Norrish AR, McRae CU, Cohen RJ, Jackson RT (1999). A population-based study of clinical and pathological prognostic characteristics of men with familial and sporadic prostate cancer. BJU Int.

[22] Heijnsdijk EA, Wever EM, Auvinen A, Hugosson J, Ciatto S, Nelen V, Kwiatkowski M, Villers A, Páez A, Moss SM, Zappa M, Tammela TL, Mäkinen T, Carlsson S, Korfage IJ, Essink-Bot ML, Otto SJ, Draisma G, Bangma CH, Roobol MJ, Schröder FH, de Koning HJ (2012). Quality-of-life effects of prostate specific antigen screening. N Engl J Med.

[23] Booth N, Rissanen P, Tammela TL, Määttänen L, Taari K, Auvinen A (2014). Health-related quality of life in the Finnish trial of screening for prostate cancer. Eur Urol.

[24] Walsh PM, McCarron P, Middleton RJ, Comber H, Gavin AT, Murray L (2006). Influence of mammographic screening on trends in breast-conserving surgery in Ireland. Eur J Cancer Prev.

[25] Hervouet S, Savard J, Simard S, Ivers H, Laverdière J, Vigneault E, Fradet Y, Lacombe L (2005). Psychological functioning associated with prostate cancer: cross-sectional comparison of patients treated with radiotherapy, brachytherapy, or surgery. J Pain Symptom Manag.

[26] Punnen S, Cowan JE, Dunn LB, Shumay DM, Carroll PR, Cooperberg MR (2013). A longitudinal study of anxiety, depression and stress as predictors of sexual and urinary quality of life in men with prostate cancer. BJU Int.

[27] Sharpley CF, Christie DR, Bitsika V (2010). Variability in anxiety and depression over time following diagnosis in patients with prostate cancer. J Psychosoc Oncol.

[28] Watts S, Leydon G, Birch B, Prescott P, Lai L, Eardley S, Lewith G (2014). Depression and anxiety in prostate cancer: a systematic review and meta-analysis of prevalence rates. BMJ Open.

[29] Bennett G, Badger TA (2005). Depression in men with prostate cancer. Oncol Nurs Forum.

[30] Sharpley CF, Bitsika V, Christie DR (2009). Understanding the causes of depression among prostate cancer patients: development of the Effects of Prostate Cancer on Lifestyle Questionnaire. Psychooncology.

[31] Brookhart MA, Patrick AR, Dormuth C, Avorn J, Shrank W, Cadarette SM, Solomon DH (2007). Adherence to lipid-lowering therapy and the use of preventive health services: an investigation of the healthy user effect. Am J Epidemiol.

[32] Drummond FJ, Flahavan EM, Bennett K, Barron TI, Sharp L (2014). Prostate specific antigen testing is associated with men’s psychological and physical health and their healthcare utilisation in a nationally representative sample: a cross-sectional study. BMC Fam Pract.

[33] Kotwal AA, Schumm P, Mohile SG, Dale W (2012). The influence of stress, depression, and anxiety on PSA screening rates in a nationally representative sample. Med Care.

[34] Consedine NS, Adjei BA, Ramirez PM, McKiernan JM (2008). An object lesson: source determines the relations that trait anxiety, prostate cancer worry, and screening fear hold with prostate screening frequency. Cancer Epidemiol Biomark Prev.

[35] Dale W, Bilir P, Han M, Meltzer D (2005). The role of anxiety in prostate carcinoma: a structured review of the literature. Cancer.

[36] Ransohoff DF, McNaughton Collins M, Fowler FJ (2002). Why is prostate cancer screening so common when the evidence is so uncertain? A system without negative feedback. Am J Med.

[37] Curtis R, Groarke A, Sullivan F (2014). Stress and self-efficacy predict psychological adjustment at diagnosis of prostate cancer. Sci Rep.

[38] Wootten AC, Burney S, Foroudi F, Frydenberg M, Coleman G, Ng KT (2007). Psychological adjustment of survivors of localised prostate cancer: investigating the role of dyadic adjustment, cognitive appraisal and coping style. Psycho-Oncology.

[39] Folkman S, Greer S (2000). Promoting psychological well-being in the face of serious illness: when theory, research and practice inform each other. Psycho-Oncology.

[40] Ben-Tovim DI, Dougherty ML, Stapleton AM, Pinnock CB (2002). Coping with prostate cancer: a quantitative analysis using a new instrument, the centre for clinical excellence in urological research coping with cancer instrument. Urology.

[41] Couper JW, Love AW, Duchesne GM, Bloch S, Macvean M, Dunai JV, Scealy M, Costello A, Kissane DW (2010). Predictors of psychosocial stress 12 months after diagnosis with early and advanced prostate cancer. Med J Aust.

[42] Hanly N, Mireskandari S, Juraskova I (2014). The struggle towards ’the new normal’: a qualitative insight into psychosexual adjustment to prostate cancer. BMC Urol.

[43] Newby TA, Graff JN, Ganzini LK, McDonagh MS (2015). Interventions that may reduce depressive symptoms among prostate cancer patients: a systematic review and meta-analysis. Psychooncology.

[44] Gallagher D, O’Regan C, Savva GM, Cronin H, Lawlor BA, Kenny RA (2012). Depression, anxiety and cardiovascular disease: which symptoms are associated with increased risk in community dwelling older adults?. J Affect Disord.

[45] Rauscher GH, Johnson TP, Cho YI, Walk JA (2008). Accuracy of self-reported cancer-screening histories: a meta-analysis. Cancer Epidemiol Biomark Prev.

