# Supplementary material for: Mode of prostate cancer detection is associated with the psychological wellbeing of survivors: results from the PiCTure study
Source: Support Care Cancer. 2015 Nov 23;24(5):2297–307. doi: 10.1007/s00520-015-3033-x (PMC4805717; doi:10.1007/s00520-015-3033-x)
Supplement: Supplementary file 1 — (DOCX 15 kb) [file 520_2015_3033_MOESM1_ESM.docx]

### Supplementary Table 1: Characteristics of prostate cancer populations: all survivors, random sample, eligible survivors, responders and non-responders

|  |  | **All survivors** | **Random sample** | **Eligible for study** | **Responders** | **Non-responders** |
| --- | --- | --- | --- | --- | --- | --- |
| Total |  | N=22,823 | N=12,322 | N=6,559 | N=3,348 | N=3,211 |
|  |  | Number and % of total survivors in each column | | | |  |
| Age at diagnosis (years) | 0-59 | 5,046 (22%) | 2,039 (17%) | 1,329 (20%) | 799 (24%) | 530 (17%) |
|  | 60-69 | 10,212 (45%) | 4,891 (40%) | 2,939 (45%) | 1,631 (49%) | 1,308 (41%) |
|  | 70+ | 7,565 (33%) | 5,392 (44%) | 2,291 (35%) | 918 (27%) | 1,373 (43%) |
| Time since diagnosis (years) | 2-5 | 9,569 (42%) | 5,340 (43%) | 3,101 (47%) | 1,614 (48%) | 1,487 (46%) |
|  | 5-9·9 | 9,776 (43%) | 4,324 (35%) | 2,114 (32%) | 1,075 (32%) | 1,039 (32%) |
|  | ≥10 | 3,478 (15%) | 2,658 (22%) | 1,344 (21%) | 659 (20%) | 685 (21%) |
| TNM stage* | I/II | 12,761 (56%) | 5,792 (47%) | 3,817 (58%) | 2,126 (64%) | 1,691 (53%) |
|  | III | 2,122 (9%) | 1,130 (9%) | 947 (14%) | 612 (18%) | 335 (10%) |
|  | IV | 690 (3%) | 445 (4%) | 267 (4%) | 141 (4%) | 126 (4%) |
|  | Unknown | 7,250 (32%) | 4,955 (40%) | 1,528 (23%) | 469 (14%) | 1,059 (33%) |
| Gleason grade^a^ | 2-4 | 1,578 (7%) | 923 (8%) | 472 (7%) | 212 (6%) | 260 (8%) |
|  | 5-7 | 11,766 (52%) | 4,996 (41%) | 3,609 (55%) | 2,186 (65%) | 1,423 (44%) |
|  | 8-10 | 2,865 (13%) | 1,594 (13%) | 1,060 (16%) | 625 (19%) | 435 (14%) |
|  | Unknown | 6,614 (29%) | 4,809 (39%) | 1,418 (22%) | 325 (10%) | 1,093 (34%) |

*Further information was sourced for TNM stage and Gleason grade for NI responders only. This is reflected in the lower percentage with stage unknown among responders compared to the other columns
